# Myxedema Coma Leading to Acute Coronary Syndrome: A Case Report

**DOI:** 10.7759/cureus.84324

**Published:** 2025-05-18

**Authors:** Amir Joshi, Warren Fernandes, Aishwarya Yamparala, Adit M Patel, Saroj K Jha, Nitish Sharma, Richard Wholey

**Affiliations:** 1 Internal Medicine, Saint Vincent Hospital, UMass Chan Medical School, Worcester, USA; 2 Internal Medicine, Tribhuvan University Teaching Hospital, Kathmandu, NPL; 3 Cardiovascular Medicine, Saint Vincent Hospital, UMass Chan Medical School, Worcester, USA

**Keywords:** acute coronary syndrome, case report, hypothyroidism, myocardial infarction, myxedema coma

## Abstract

Myxedema coma is a rare and life-threatening medical condition. We present a case of poorly controlled hypothyroidism that initially caused myxedema coma and then led to acute coronary syndrome (ACS).

A 57-year-old woman with a history of Hashimoto's thyroiditis and coronary artery bypass grafting (CABG) came in with fatigue and worsening left-sided chest pain that occurred even at rest for the past 12 hours. She has not been taking her levothyroxine as prescribed and has not been seeing her endocrinologist for follow-up. When she arrived, her vitals showed that she had a heart rate of 53 beats per minute (sinus bradycardia), but otherwise, she was stable.

The laboratory tests showed elevated levels of high sensitivity troponin T at 42 ng/L (normal value: <14 ng/L), a thyroid stimulating hormone level of 408 mIU/L (normal value: 0.5-2.5 mIU/L), a free thyroxine level of 0.4 (normal range: 0.8-1.8 ng/mL), and a decreased glomerular filtration rate of 71 mL/min/1.73 m^2^ (normal range: 90-120 mL/min/1.73 m^2^). Although the electrocardiogram did not show ST-T wave changes, the thrombolysis in myocardial infarction (TIMI) risk score was five. Additionally, there was a new onset decrease in ejection fraction to 40% and mild hypokinesia of the left ventricle on the echocardiogram.

She was started on a heparin drip in the emergency department and subsequently underwent cardiac catheterization with drug-eluting stent (DES) placement. Myxedema coma score was 40 suggestive of coma risk myxedema and eventually, she was admitted to the intensive care unit. Her condition was managed using intravenous levothyroxine, liothyronine, and hydrocortisone. After her symptoms subsided, she was discharged with a prescription for dual antiplatelet agents and levothyroxine.

Due to its rarity and high mortality rate, it is crucial for physicians to maintain a high level of suspicion for myxedema coma and promptly initiate treatment. This is especially important when a patient with a history of hypothyroidism presents with cardiac issues such as ACS or bradycardia that do not entirely align with the clinical picture.

## Introduction

Myxedema coma is a rare and critical medical emergency that represents the most severe form of hypothyroidism, with an estimated incidence of 0.22 per million annually in the general population [1]. Myxedema coma is characterized by hypothermia, altered mental status, bradycardia, hypotension, and multi-organ dysfunction, secondary to long-standing hypothyroidism. The cardiovascular system is particularly vulnerable to the metabolic and hemodynamic changes seen in hypothyroidism, with potential manifestations including decreased cardiac output, pericardial effusion, and conduction abnormalities such as bradycardia [2]. Patients with known stable angina may experience fewer symptoms during a hypothyroid state due to decreased activity and lower oxygen demand. Hypothyroidism has been linked to accelerated coronary artery disease (CAD) [3]. However, myxedema coma rarely leads to acute coronary syndrome (ACS) [4].

This case report details an unusual presentation of myxedema coma that initially manifested as ACS. It highlights the importance of considering endocrine abnormalities, particularly hypothyroidism, in patients presenting with anginal chest pain, especially when there is a history of hypothyroidism and poor adherence to treatment.

## Case presentation

A 57-year-old woman presented to the emergency department with complaints of worsening fatigue and left-sided chest pain for 12 hours. The chest pain was described as a dull, constant pressure that radiated to her left shoulder, which occurred even at rest. She also reported mild shortness of breath but denied any nausea, diaphoresis, palpitations, or loss of consciousness. She also stated about increased fatigue, dry skin, and cold intolerance over the past several months, which she attributed to aging. Her past history is significant for hypertension, hyperlipidemia, Hashimoto’s thyroiditis, CAD, and coronary artery bypass grafting (CABG) done five years ago. She was not adherent to levothyroxine therapy for more than a year and had not followed up with an endocrinologist. She does not drink alcohol but smoked cigarettes in the past.

On presentation, the patient was alert but appeared lethargic. Her vital signs revealed bradycardia with a heart rate of 53 beats per minute (bpm), blood pressure of 102/68 mmHg, respiratory rate of 18 breaths per minute, and temperature of 35.8°C (96.4°F). Her oxygen saturation was 96% on room air. Physical examination revealed dry, coarse skin, thinning eyebrows, periorbital puffiness, and non-pitting edema in her lower extremities. Respiratory and cardiovascular examinations were within normal limits. Her Glasgow Coma Scale (GCS) score was 15. Cranial nerve examinations, motor examinations, and sensory examinations were all grossly intact. Deep tendon reflexes were sluggish.

**Figure 1:**
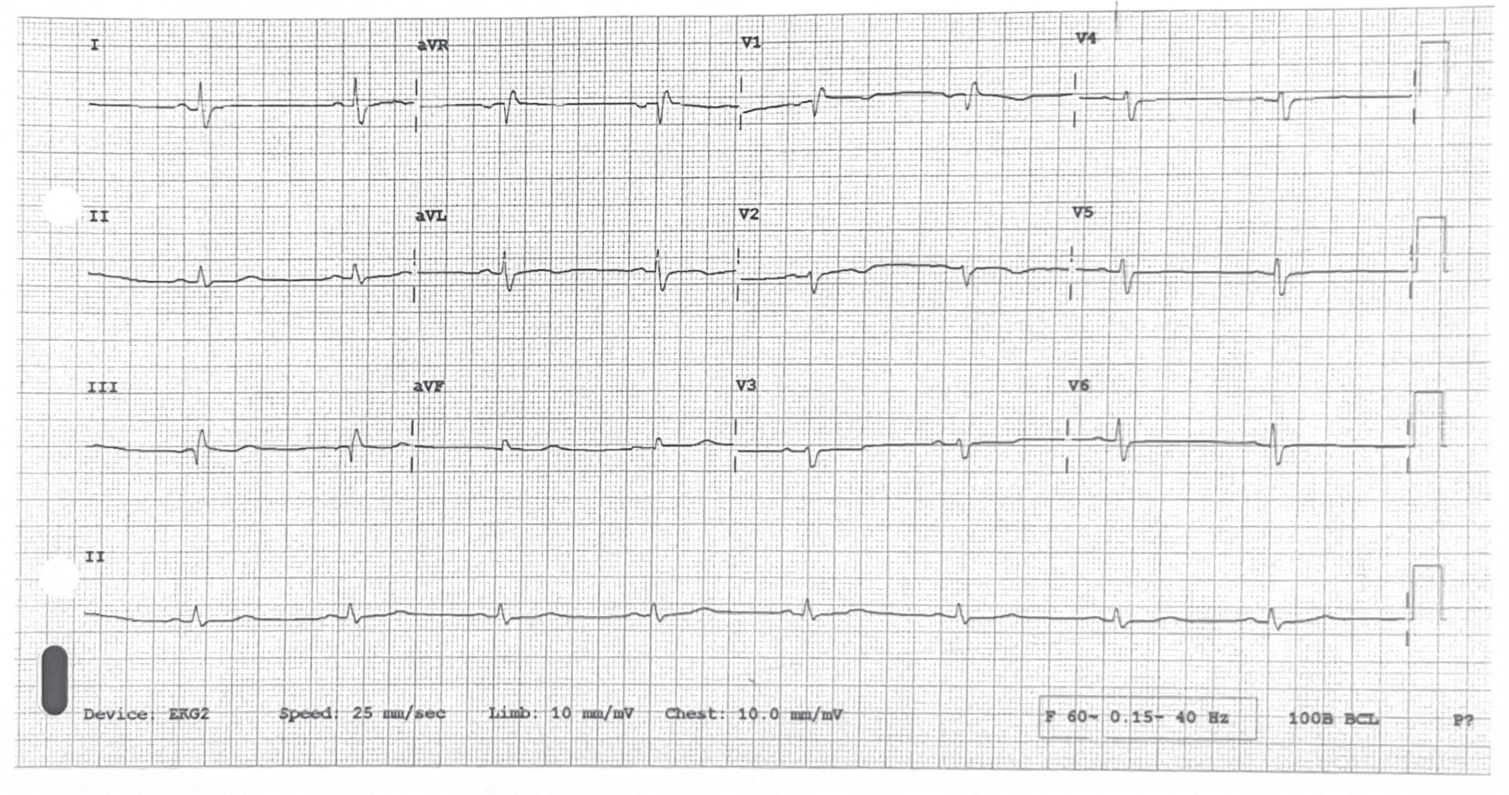
Admission ECG showing bradycardia ECG: electrocardiogram

Given her symptoms and medical history, initial concern was raised for ACS. Initial laboratory investigations, done at 12 hours of symptom onset, i.e., at the time of admission are given in Table 1. An electrocardiogram (ECG) showed sinus bradycardia without significant ST-T wave changes (Figure 1). However, laboratory results showed an elevated high-sensitivity troponin T level of 42 ng/L (reference range: <14 ng/L), suggesting myocardial infarction. Chest X-ray was unremarkable. The echocardiogram showed mild hypokinesia of the left ventricle with a new reduction in ejection fraction to 40% (down from 55% two years ago). Further investigations revealed profound hypothyroidism with a thyroid-stimulating hormone (TSH) level of 408 mIU/L (reference range: 0.5-2.5 mIU/L) and free thyroxine (T4) level of 0.4 ng/dL (reference range: 0.8-1.8 ng/dL). Her serum cortisol was within the normal range, and a basic metabolic panel showed a reduced glomerular filtration rate (GFR) of 71 mL/min/1.73 m², suggestive of mild renal impairment.

**Table 1:** Laboratory values on admission BUN: blood urea nitrogen; TSH: thyroid-stimulating hormone; fT4: free thyroxine; hs-TnT: high-sensitivity troponin T

Parameters	Result	Reference value
WBC	8.9 × 10^9^/mm^3^	4.0-10.0 × 10^9^/mm^3^
Hemoglobin	14.1 g/dL	11.6-15 g/dL
Hematocrit	42.8%	35.5-44.9%
Platelets	282 × 10^9^/mm^3^	157-371 × 10^9^/mm^3^
BUN	21	6-21 mg/dL
Creatinine	0.85 mg/dL	0.5-1.1 mg/dL
Sodium	135 mEq/L	135-145 mEq/L
Potassium	4.1 mEq/L	3.5-5.0 mEq/L
CO_2_	23 mEq/L	23-29 mEq/L
Glucose	96 mg/dL	≤200 mg/dL
Anion gap	13	4-12
TSH	408 mIU/L	0.5-2.5 mIU/L
fT4	0.4 ng/dL	0.8-1.8 ng/dL
hs-TnT	42 ng/L	<14 ng/L

**Figure 2:**
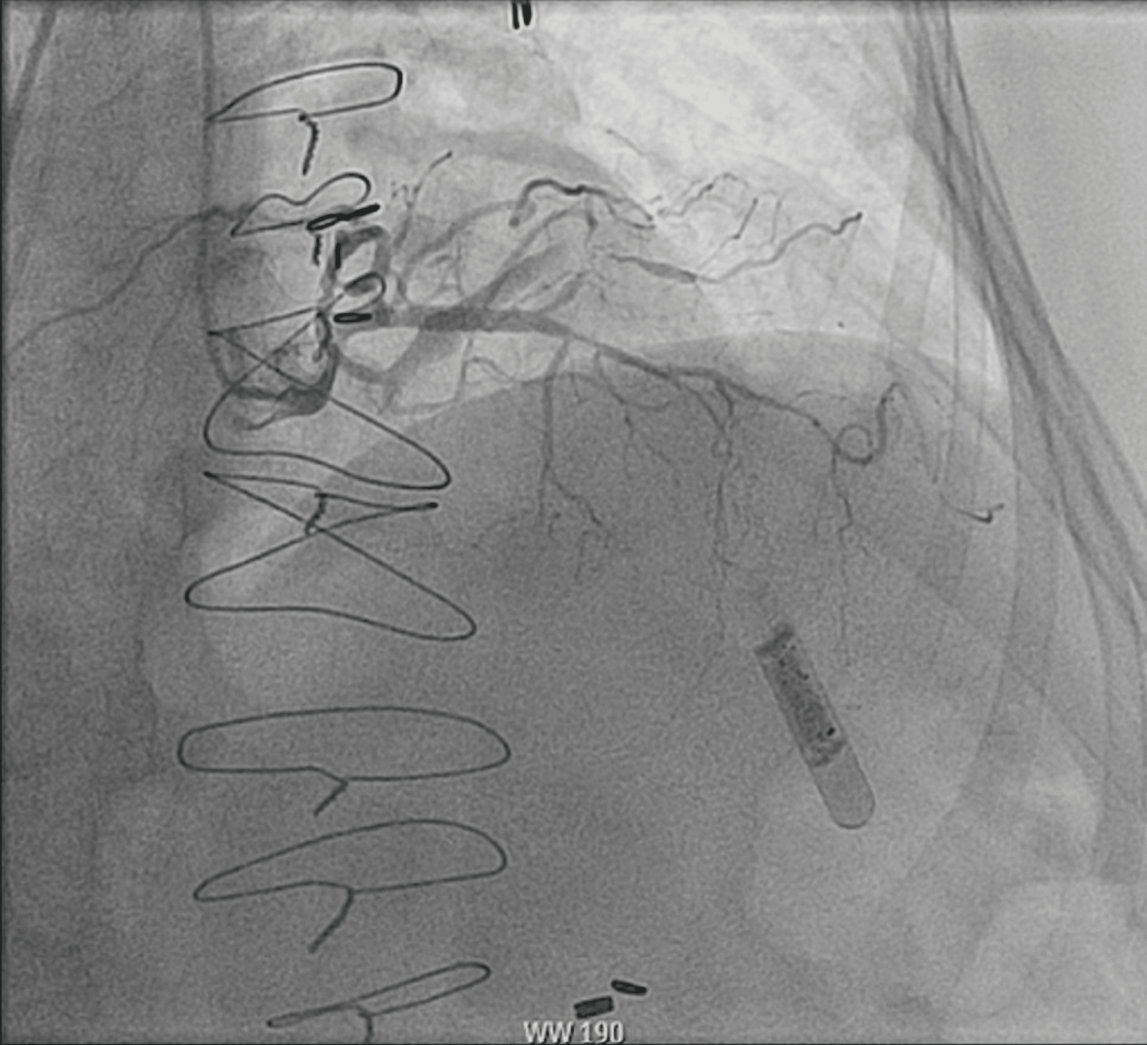
Coronary angiogram showing 99% stenosis in D2

**Figure 3:**
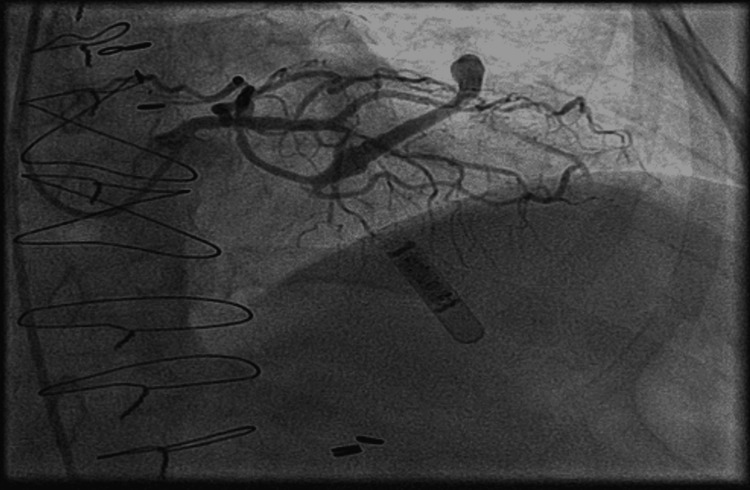
Coronary angiogram after DES placement DES: drug-eluting stent

Although the ECG showed no ST-T wave changes, the rise in creatinine and a new decrease in ejection fraction with mild left ventricular hypokinesia was concerning for possible ACS. She had a thrombolysis in myocardial infarction (TIMI) risk score of five. The patient was initiated on a heparin drip for possible ACS. Aspirin 325 mg, atorvastatin 80 mg, and a loading dose of ticagrelor were also given. Cardiac catheterization revealed 99% stenosis of the second diagonal artery D2 (Figure 2). Drug-eluting stent (DES) was placed which relieved the obstruction (Figure 3). Then she was transferred to the intensive care unit (ICU). The myxedema coma score was 40. Therefore, she received intravenous levothyroxine 100 mcg, liothyronine, and hydrocortisone.

Her bradycardia and lethargy gradually improved with thyroid hormone replacement therapy. She was hemodynamically stable. The patient was transitioned to oral levothyroxine. She was instructed to strictly adhere to her thyroid hormone replacement regimen and dual antiplatelet therapy (aspirin and clopidogrel) for cardiovascular protection. A follow-up with endocrinology and cardiology was scheduled.

## Discussion

Myxedema coma is a critical condition originating from severe thyroid dysfunction, resulting in the gradual failure of multiple organ systems and posing a significant risk of death. This extreme form of hypothyroidism is characterized by low blood pressure, slow heart rate, altered mental state, low sodium levels, and hypoglycemia [4]. It often presents with non-specific symptoms that mimic various medical emergencies like heart failure and septic shock. However, the overlap with ACS is rare.

Thyroid hormones play a crucial role in maintaining cardiovascular homeostasis, including regulating heart rate, myocardial contractility, and vascular resistance. The primary cardiovascular manifestations of hypothyroidism include bradycardia, reduced cardiac output, increased systemic vascular resistance, and diastolic hypertension [5]. In more severe cases, like myxedema coma, these effects are exacerbated, leading to profound hemodynamic compromise [6]. The patient in this case presented with a constellation of cardiovascular symptoms, including bradycardia (heart rate of 53 bpm) and a reduced left ventricular ejection fraction (40%), which are consistent with the effects of thyroid hormone deficiency on cardiac function. These findings, along with the elevated troponin levels and the new left ventricular hypokinesia, raised suspicion for ACS. However, the underlying severe hypothyroidism likely played a significant role in exacerbating her cardiovascular instability.

In the case of hypothyroidism, the heart experiences a reduction in beta-adrenergic receptor activity, leading to decreased heart rate and contractility [7]. Several mechanisms explain how hypothyroidism and myxedema coma may precipitate or worsen ACS. First, hypothyroidism leads to hyperlipidemia, primarily by reducing the clearance of low-density lipoprotein (LDL) cholesterol and triglycerides, increasing the risk of atherosclerotic plaque formation [8,9]. The patient's history of hyperlipidemia and CAD likely contributed to the stenosis of her coronary artery, which was revealed during cardiac catheterization. Second, hypothyroidism is associated with diastolic hypertension, which increases the afterload on the left ventricle, further exacerbating ischemic conditions in patients with pre-existing coronary artery stenosis [8]. This increased afterload, combined with the reduced contractility and bradycardia seen in myxedema coma, reduces myocardial oxygen delivery while increasing myocardial oxygen demand, thus contributing to myocardial ischemia and infarction. Moreover, hypothyroidism may impair coronary blood flow through direct effects on vascular smooth muscle and endothelial function, leading to increased arterial stiffness and reduced coronary perfusion [5,10]. This patient’s significant coronary artery stenosis (99% in the second diagonal artery) was the most likely cause of her ACS, but the severe hypothyroidism may have played a role in exacerbating her ischemic symptoms.

One of the major diagnostic challenges in this case was distinguishing between ACS as the primary pathology and myxedema coma as the precipitating factor. The patient’s clinical presentation with chest pain, bradycardia, and elevated troponin levels initially raised suspicion for ACS, which prompted an urgent cardiology evaluation and initiation of anticoagulation. However, the profound hypothyroidism and non-specific symptoms such as fatigue, dry skin, and cold intolerance should have also raised suspicion for myxedema coma, especially given her non-adherence to levothyroxine therapy.

The diagnosis of myxedema coma is largely clinical, supported by laboratory findings of profound hypothyroidism (elevated TSH and low free T4) and clinical features such as altered mental status, bradycardia, and hypothermia. In this case, the patient’s TSH level of 408 mIU/L and free T4 level of 0.4 ng/dL were consistent with severe hypothyroidism. Although the patient’s mental status was not severely altered, her lethargy and sluggish responses were suggestive of early myxedema coma. It is important to note that in some cases of myxedema coma, cardiovascular symptoms such as chest pain and bradycardia may be the predominant presenting features, further complicating the diagnostic process [11,12]. This overlap underscores the need for a thorough evaluation of thyroid function in patients presenting with cardiovascular symptoms, especially in those with a known history of hypothyroidism.

The management of myxedema coma requires prompt recognition and initiation of thyroid hormone replacement, in addition to supportive care for any co-existing conditions such as ACS. In this case, the patient’s ACS was managed with cardiac catheterization and placement of a DES to relieve the 99% stenosis of the second diagonal artery. The prompt restoration of coronary blood flow was critical in stabilizing her cardiovascular status. Simultaneously, the patient’s myxedema coma was treated with intravenous levothyroxine and liothyronine to rapidly restore thyroid hormone levels. Given the severity of her hypothyroidism, intravenous therapy was preferred over oral replacement to ensure adequate absorption and bioavailability. Additionally, the administration of hydrocortisone was essential to prevent adrenal insufficiency, which can occur in patients with severe hypothyroidism, particularly in the setting of stress or critical illness [13,14]. Early intervention with thyroid hormone replacement in patients with myxedema coma can significantly improve prognosis, even in those with concomitant ACS [15].

The prognosis of myxedema coma largely depends on the timeliness of diagnosis and the rapidity with which thyroid hormone replacement is initiated [16]. In this case, the patient’s prompt transfer to the ICU and initiation of intravenous levothyroxine, liothyronine, and hydrocortisone were key factors in her recovery. However, even with appropriate treatment, the mortality rate of myxedema coma remains high, ranging from 30% to 60% [17], emphasizing the need for early recognition and aggressive management. Long-term management of patients who have experienced myxedema coma requires strict adherence to thyroid hormone replacement therapy, regular monitoring of thyroid function, and follow-up with endocrinology to ensure adequate control of hypothyroidism [18]. In addition, patients with co-existing cardiovascular disease, like this patient, must be closely followed by cardiology to optimize secondary prevention strategies, including antiplatelet therapy, statin therapy, and control of hypertension and hyperlipidemia.

## Conclusions

Myxedema coma is a medical emergency that requires prompt diagnosis and treatment. It can present simultaneously with symptoms mimicking other serious conditions, such as ACS, which requires prompt intervention to mitigate the risk of mortality. Therefore, it is essential for physicians to maintain a high index of suspicion in patients with known hypothyroidism. The case underscores the importance of considering endocrine causes when evaluating patients with cardiovascular symptoms and highlights the need for thorough history-taking and comprehensive diagnostic workup. Timely intervention with thyroid hormone replacement therapy can significantly improve outcomes in patients with myxedema coma. Compliance with thyroid replacement therapy improves long-term prognosis and prevents cardiac complications of hypothyroidism. This case report highlights the importance of early recognition of the symptoms of myxedema coma in the setting of cardiovascular instability and emphasizes the need for a multidisciplinary approach in managing both thyroid dysfunction and ACS.
